# Outbreak report of investigation and control of an outbreak of Panton-Valentine Leukocidin-positive methicillin-sensitive *Staphylococcus aureus* (PVL-MSSA) infection in neonates and mothers

**DOI:** 10.1186/s12879-019-3802-0

**Published:** 2019-02-20

**Authors:** G. Gopal Rao, R. Batura, R. Nicholl, F. Coogan, B. Patel, P. Bassett, A. M. Kearns

**Affiliations:** 1 Department of Microbiology, Paediatrics and Infection Control, London North West Healthcare, London, UK; 2 Faculty of Medicine, Imperial College, London, UK; 3 Neonatal Unit, London North West Healthcare, London, UK; 4 Public Health, England, UK; 5 Statsconsultancy Ltd, London, UK; 6 Antimicrobial Resistance and Healthcare Associated Infections Reference Unit, National Infection Service, Public Health England, London, UK

**Keywords:** PVL-MSSA, Outbreak, Neonatal unit

## Abstract

**Background:**

In January 2011, there was an outbreak of Panton-Valentine Leukocidin-positive methicillin-sensitive *Staphylococcus aureus* (PVL-MSSA) infection in a neonatal unit (NNU).

We describe the investigation and control of an outbreak of PVL-MSSA infection in neonates. Setting: Neonatal unit in West London.

**Methods:**

We performed descriptive and analytical (case-control study) epidemiological investigations. Microbiological investigations including screening of MSSA isolates by PCR for the presence of the *luk-PV*, *mecA* and *mecC* genes and comparison of isolate with Pulsed field gel electrophoresis (PFGE). Control measures were also introduced.

**Results:**

Sixteen babies were infected/colonised with the outbreak strain. Of these, one baby developed blood stream infection, 12 developed skin pustules and four babies were colonised.

Four mothers developed breast abscesses. Eighty-seven babies in the unit were screened and 16 were found to have same PVL-MSSA strain (*spa* type t005, belonging to MLST clonal complex 22). Multivariate analysis showed gestational age was significantly lower in cases compared to controls (mean gestational age: 31.7 weeks v 35.6 weeks; *P* = 0.006). Length of stay was significantly greater for cases, with a median of 25 days, compared to only 6 days for controls (*P* = 0.01). Most (88%) cases were born through caesarean section, compared to less than half of controls. (*P* = 0.002).

No healthcare worker carriers and environmental source was identified. The outbreak was controlled by stopping new admissions to unit and reinforcing infection control precautions. The outbreak lasted for seven weeks. No further cases were reported in the following year.

**Conclusions:**

Infection control teams have to be vigilant for rising prevalence of particular *S. aureus* clones in their local community as they may cause outbreaks in vulnerable populations in healthcare settings such as NNUs.

## Strengths of the study

- This would be the first published detailed report of investigation and successful control of a PVL-MSSA outbreak in a neonatal unit in the UK.
- The report also describes implications for breast feeding mothers.

## Limitations of the study

- This study is limited to a single unit and the description of the spread and control of the outbreak may not be generalizable
- As mothers of babies admitted to the unit were not followed up actively in the community, the extend of spread in the community could not be determined
- We were not able to confirm the time and source of initial introduction of the outbreak strain

## Background

In 1932, Panton and Valentine described a toxin with leukocidal activity (now known as Panton-Valentine leukocidin) produced by some strains of *S.aureus*.^1^ In the 1950s and 1960s, a strain of PVL-MSSA (phage type 80/81, now known to belong to multilocus sequence typing (MLST) clonal complex 30) was associated with disease and widely distributed in community and healthcare settings in the UK and abroad [1]. Recent research has shown that virulence factors such as *agr* and *hla* along with penicillin resistance contributed to the epidemic potential of the phage type 80/81 clone [2]. The development of methicillin and other penicillinase resistant penicillins in 1960s may have successfully contributed to the observed decline in this successful lineage [3]. Genetically diverse PVL-positive MSSA and MRSA clones have emerged worldwide [4]. PVL-MSSA causes the same spectrum of clinical disease as PVL-MRSA and has similar epidemiological characteristics [5]. They are associated with skin and soft tissue infections (SSTIs) but can also cause life threatening disease such as necrotising pneumonia and necrotising fasciitis [6]. Following the first report of community associated PVL-MRSA in four children in North Dakota in the United States in 1999, there have been numerous reports of outbreaks in neonatal units (NNUs) in various countries including the UK [7–10]. This is probably because of the perceived public health importance of these strains owing to their resistance to methicillin and potential to cause life threatening infection in previously healthy individuals in the community.

Compared to PVL-MRSA, there are relatively few reports of outbreaks of PVL-MSSA infection in NNUs despite such strains being more prevalent than PVL-MRSA in the UK and continental Europe [11, 12]. At the time of the outbreak, there had been a tenfold increase in the number cases of PVL-SA identified by the national Staphylococcus Reference Unit in England over a six year period (rising from 224 in 2005 to 2227 in 2010), 58% of which were MSSA.

In this report, we describe the investigation and management of an outbreak of PVL-MSSA in a NNU in a large general hospital in North West London, UK.

### Aim, design and setting

North West London Healthcare serves the population of the boroughs of Brent and Harrow in London, UK. The NNU at London North West Healthcare is organized in three nurseries and an Intensive Therapy Unit with eight cots each.

### Characteristics of participants

Newborns admitted to the NNU are low birth-weight (< 2500 g), preterm infants or in a clinically unstable condition. At the time of admission to the unit, neonates are screened for any infection or asymptomatic carriage of organisms of interest for infection control purposes such as MRSA and multidrug resistant gram negative bacilli by culture of throat, rectal and umbilical swabs. Babies transferred from other hospitals are also screened for MRSA on admission. Outbreaks of infection or suspected transmission events are detected by clinical observation and laboratory-based surveillance.

## Methods

**Fig. 1:**
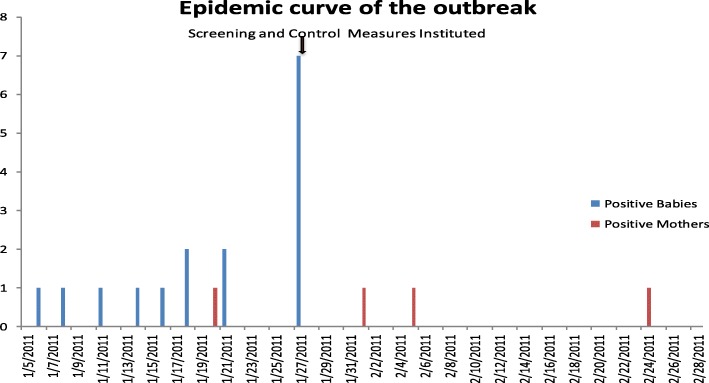
Epidemic Curve of the outbreak in neonatal Unit

Investigation of this outbreak was triggered by detection of PVL-MSSA from a blood culture taken from a pre-term baby (gestational age of 27 weeks) in the NNU on 07/01/2011. Over the next two weeks till 21/01/2017, clinicians in the NNU alerted the infection control team about an unusual number of babies with skin infection (pustules) and one mother of the babies in the unit developed a breast abscess. Following the recovery of MSSA from skin pustules, an outbreak was declared on and control measures instituted on 27/01/2011. After the institution of control measures, no further cases were detected among the babies but three further cases of breast infection were observed in mothers of the babies in the unit (Fig. 1).

During the outbreak period (7th January – 28th February 2011), MSSA were recovered from specimens (clinical and screening) from 20 neonates and four mothers who developed breast abscesses by culturing on blood agar (Thermo Fisher Scientific, UK) and incubating in CO_2_ at 37 °C for 18–24 h. Antibiotic susceptibility to cefoxitin and other antibiotics was performed using the methods recommended by British Society for Antimicrobial Chemotherapy [13] MSSA strains were sent to the national reference laboratory (Public Health England, Colindale, London) for characterisation. The isolates were screened by PCR for the presence of the *luk-PV*, *mecA* and *mecC* genes [14]. These isolates were further characterised by *spa* typing and pulsed-field gel electrophoresis (PFGE) as described previously [5]. Isolates were subsequently assigned a presumptive MLST clonal complex based on spa typing data and by reference to spa server (http://spa.ridom.de/mlst.shtml) and MLST (http://saureus.mlst.net) databases.”

Throat and nose of healthcare and allied workers in the maternity and neonatal units were screened for the outbreak strain. The mothers of the babies were not screened. Environmental specimens collected at the time of the outbreak from obstetric theatres, incubators, diagnostic equipment and the breast milk expression machines and frequently touched surfaces in the NNU and maternity unit were also tested for the outbreak strain or any other MSSA.

**Fig. 2:**
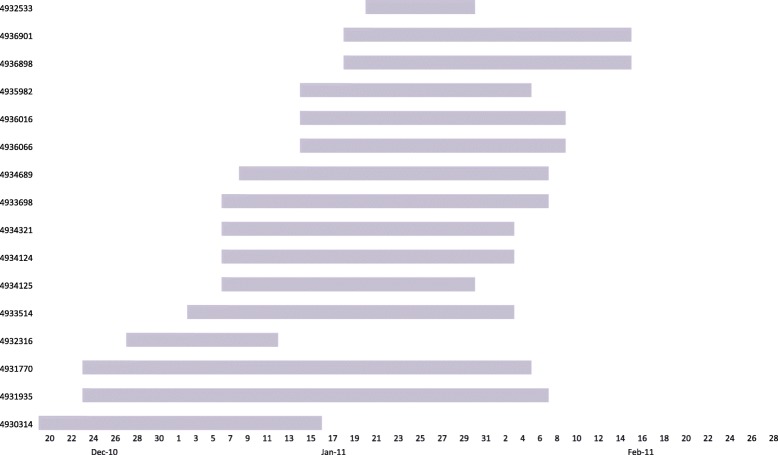
Timeline of the outbreak showing dates of admission and discharge to the neonatal unit

Sixteen babies were infected/colonised with the outbreak strain. The first infected/colonised neonate was detected on 07 Jan 2011 and the last neonate on 27 Jan 2011. Of the 16, one baby developed bacteraemia, 11 babies developed skin pustules and four babies were colonised with the outbreak strain. Four mothers developed breast abscesses. The first mother with breast abscess was detected on 11th January and the last infected mother was detected on 24th February 2011. The outbreak period was defined as 07 Jan 2011 to 28 Feb 2011, four weeks after the last infected/colonised neonate. The epidemic curve of the outbreak is shown in Fig. 1 and the timeline in Fig. 2. During the outbreak period, 87 babies were admitted to the NNU.

A case-control study was carried out. Babies colonised/infected with outbreak strain and babies without the outbreak strain were classified as cases and controls respectively. The initial analysis compared the characteristics of cases and controls.

### Statistical analysis

Continuous variables were analysed using the unpaired t-test where found to be normally distributed; where the values did not follow a normal distribution, the Mann-Whitney test was used. Categorical variables were compared between cases and controls using Fisher’s exact test.

Subsequently the joint association between patient characteristics and case/control status was examined in a multiple logistic regression analysis. Due to the relatively small number of cases, only factors found to significantly differ between groups from the initial analyses were included in the analysis. A backwards selection procedure was performed to retain only those variables that were found to significantly influence the outcome. This involved removing non-significant variables, one at a time, until only significant variables were left.

## Results

Twenty babies were found to be colonised with MSSA. Of these, 19 were PVL-MSSA. *Spa* typing data showed *spa* type t005 belonging to MLST-CC 22 was predominant (*n* = 16) and this group of strains were indistinguishable by PFGE. The remainder comprised 3 distinct PVL-SA strains t008 (*n* = 1), t127 (*n* = 1), t393 (*n* = 1) belonging to MLST clonal complexes 8, 1 and 15 respectively. The PVL negative MSSA strain belonged to spa type t002. Pus from the breast abscesses of four mothers yielded *spa* type t005 PVL-MSSA which were indistinguishable from the outbreak strain. The outbreak strain was resistant to penicillin and gentamicin but susceptible to flucloxacillin, erythromycin, clindamycin, fucidin, rifampicin, amikacin, vancomycin and teicoplanin.

Twelve of 20 babies with MSSA had skin pustules. The outbreak strain was identified from16/20 babies (16.5%, 9 females, 7 males). One baby (index case) developed presumptive haemorrhagic pneumonia and died. Due to the extreme prematurity of the baby (27 weeks) and because no post mortem was performed, it was not clear if PVL-MSSA was the cause of pneumonia or death. None of the healthcare and allied workers in the maternity and neonatal units were found to carry the outbreak strain. All environmental specimens were also negative.

Summary statistics of the case control study for possible risk factors along with *p*-values indicating the significance of the results are shown in Table 1.

**Table 1:** Case Control Study: Univariate analysis of risk factors

Variable	Category	Controls (*n* = 81)	Cases (*n* = 16)	*P*-value
Sex	Female	34 (42%)	9 (56%)	0.41
	Male	47 (58%)	7 (44%)	
Gest age (weeks)	–	35.6 ± 4.5	31.7 ± 2.7	**0.001**
Birthweight (g)	–	2456 ± 997	1550 ± 702	**< 0.001**
Admission location	Northwick Park	72 (89%)	15 (94%)	1.00
	Other	9 (11%)	1 (6%)	
Length of stay	–	6 [1, 15]	25 [22, 29]	**< 0.001**
Mother’s ethnicity	White	22 (28%)	5 (31%)	0.73
	Asian	17 (21%)	5 (31%)	
	Black	37 (%)	6 (38%)	
	Other	4 (5%)	0 (0%)	
Mode of delivery	Vaginal	44 (55%)	2 (12%)	**0.002**
	CS	36 (45%)	14 (88%)	
Birth location	Northwick Park	73 (27%)	15 (94%)	1.00
	Other	8 (10%)	1 (6%)	

Summary statistics are mean ± standard deviation, median [inter-quartile range] or number (percentage)

*CS* Caesarean Section

The results suggest that cases and controls differed significantly. Gestational age and birth weight were significantly lower in cases compared to controls. The mean gestational age was 35.6 weeks for controls, but only 31.7 weeks for cases. Conversely, the length of stay was significantly greater for cases, with a median of 25 days, compared to only 6 days for controls.

The joint association of the variables upon case control status was examined in a multivariable analysis. A backwards selection procedure was used to retain only the statistically significant variables in the final model (Table 2).

**Table 2:** Multivariate model showing significant risk factors

Variable	Odds Ratio (95% CI)	*P*-value
Gestational age (control v cases)	0.80 (0.69, 0.94)	0.006
Caesarean Section delivery (cases v controls)	8.75 (1.69, 45.2)	0.01

The results suggest that only gestational age and mode of delivery were significantly associated with case control status. After adjusting for these variables, there were no additional effects of either birth weight or length of stay, which were significant in the initial analyses. There was a strong correlation between gestational age and birth weight, which would explain the lack of significance for this variable.

Longer gestational age was associated with a reduced likelihood of a case. Delivery by caesarean section was associated with an increased risk of being a case. The odds of being a case were over 8 times higher for babies born through caesarean section than for those with a vaginal delivery.

Both gestational age and caesarean section were significant in the multivariable analysis.

The effects of each variable were adjusted for the effects of the other variable. Whereas these variables might be correlated, this correlation was taken into account in the analysis. Despite any associations, they were both independently associated with case/control status. Both gestational age and caesarean section appear to be important risk factors.

Length of stay (LoS) was also considered as a predictor variable, and found not to be significant in the multivariable analysis. Gestational age and caesarean section were stronger predictors than LoS,

It was not possible get accurate information regarding the location of babies within the neonatal unit as many had been discharged at the time of epidemiological investigation.

### Control measures

The control measures were commenced on 27th Jan 2017. As a part of the control measures, the NNU was closed to new admissions of babies of gestational age < 34 weeks. All affected babies were isolated in a cohort bay in the neonatal unit. Babies with superficial skin infections were treated with flucloxacillin or clindamycin and made a complete recovery.

All babies, including those who did not carry the outbreak strain, received daily antimicrobial wash (Octenisan, Shulke, UK) together with a daily change of bed linen throughout the duration of hospital stay. The importance of hand hygiene and other infection control practices were frequently reinforced. An enhanced cleaning regimen was implemented and nurseries were sequentially decanted to an unoccupied nursery and fumigated once with hydrogen peroxide (Bioquell, UK). Visitors to the unit were restricted to parents or guardians. Prior to the outbreak antibiotic guidelines for empiric treatment of suspected infection in the newborns was intravenous benzyl penicillin and gentamicin; during the outbreak the latter was replaced with amikacin to which the outbreak strain was susceptible. The mothers who developed breast abscesses had breastfed their babies on the unit. Other mothers of the babies on the unit were advised to contact their GP if they suspected that were developing an infection in the breast or skin. We advised the mothers to continue breast feeding their babies as we felt that the benefits outweighed the risks. Of the four mothers who developed a breast abscess that was positive for PVL, three were recorded as having breast fed their baby.

The infections were managed by their GPs or referred to the breast surgeons. One of the mothers had surgery to manage an extensive abscess. All infected mothers were treated with clindamycin and advised antimicrobial body washes with chlorhexidine. Key information regarding the outbreak was disseminated to the staff and parents of babies in the NNU; other NNUs in the area and local GPs were also informed. Following the institution of control measures and surveillance, there were no further cases of PVL-MSSA infection in the neonatal unit in next `12 months.

## Discussion

We describe the investigation and control of an outbreak of PVL-MSSA in a neonatal unit in North West London.

There have been only two reports in the media concerning outbreaks of PVL-MSSA infections in maternity and neonatal units in England. One outbreak occurred in the maternity unit in South West England in 2003 and affected ten mothers and babies [15]. The second occurred in 2006 in a NNU in the East of England where six premature babies were affected one of whom died [16]. Sara Romano-Bertrand et al in France described an outbreak of PVL-MSSA in their neonatal unit [12]. None of these reports described the prevalence of PVL-MSSA strains in the local community. Interestingly, Tinelli and colleagues in Italy reported the association between an increase in prevalence of PVL-SA in the community and introduction of the organism into neonatal and maternity units leading to a prolonged outbreak of PVL-MSSA infection [11]. The outbreak strain belonged to the same *spa* type (t005) as the outbreak described in this incident. It is noteworthy that MSSA and MRSA representatives of the CC22 lineage have recognised epidemic and pandemic potential; this genetic background has proved adept at adapting and being successful in a range of niches from healthcare through to community settings. During the course of this investigation, PVL-MSSA strains belonging to other lineages were identified (CC1, CC8 and CC15) which have the potential to cause SSTIs in the community in England [17]. In the UK, MSSA strains from babies with infections such as pustules are not routinely tested for PVL so this level of detail may be overlooked by both clinicians and infection control teams. This incident highlights the high transmissibility associated with PVL-SA strains. Further, it is likely that transmission events especially those involving MSSA (irrespective of their PVL status) may go undetected or be associated with prolonged outbreaks prior to detection. In our investigation of the outbreak, in addition to the outbreak strain of PVL-MSSA (CC22, t005), we detected three other strains belonging to different clonal complexes, raising the possibility of a polyclonal outbreak with one predominant strain.

This outbreak demonstrates that PVL-SA may spread from the community into healthcare settings. Maternity and neonatal units appear to be particularly vulnerable to PVL-SA infections/outbreaks[11, 15]. This could be because of the close association between the mother and the baby, vulnerability of premature babies to infection, invasive procedures and frequent handling of babies by the NNU staff. We could not identify the source of the outbreak strain but the case-control study found that low gestational age, low birth weight and delivery by caesarean section were associated with the cases although these factors are likely to be inter-related. Previous reports of outbreaks have described the vulnerability of premature neonates to PVL-MSSA. It is possible that mothers or babies can acquire PVL-MSSA from each other. Indeed, mothers could be an important interface between the community and hospital.

We could not establish the mode of spread of the outbreak strain despite review of medical notes, hand hygiene and infection control practices, environmental screening and interviews with the healthcare workers in the NNU. More recent studies have shown how advanced technologies such as Whole Genome Sequencing can provide more timely, granular data for outbreak investigations, providing unprecedented insights into MRSA transmission pathways in healthcare and community settings [18].

Although no source of the outbreak was found, it was controlled by reinforcing conventional infection control measures. Following the institution of control measures and surveillance, there were no further cases of PVL-MSSA infection in the neonatal unit in next `12 months.

This highlights the critical importance of stringent infection control practice to prevent transmission of infectious agents in neonatal units.

### Limitations

As we did not undertake an active surveillance for MSSA infections before the index case described in this paper, we cannot accurately state when the outbreak started. As a result, we were unable to confirm the source of initial introduction of the outbreak strain. This study is limited to a single unit and the description of the spread and control of the outbreak may not be generalizable. As not all mothers of babies admitted to the unit were followed up actively in the community, the extend of spread in the community could not be determined.

## Conclusions

This report serves to alert clinicians in maternal and neonatal units that PVL-MSSA strains that are prevalent in the community can cause considerable morbidity in both neonates and their mothers. These infections can also lead to prolonged hospital stay. Neonatologists, obstetricians, microbiologists and infection control practitioners should be vigilant for any increase in incidence of staphylococcal infection in neonates or their mothers and should consider the possibility of PVL-MSSA and institute appropriate control measures to obviate transmission.

## Abbreviation

PVL-MSSA: Panton-Valentine Leukocidin-positive methicillin-sensitive *Staphylococcus aureus*
